# Edaravone Attenuates Sleep Deprivation-Induced Memory Deficits by Inhibiting Oxidative Stress and Neuroinflammation in Murine Models

**DOI:** 10.3390/biomedicines13051047

**Published:** 2025-04-25

**Authors:** Shiliang Ji, Yongchao Dong, Zixiang Wang, Ruifang Zhu, Yiguo Jiang, Shengjun Li, Xinwei Ma

**Affiliations:** 1 Suzhou Research Center of Medical School, Suzhou Hospital, Affiliated Hospital of Medical School, Nanjing University, Suzhou 215153, China; dg21300014@smail.nju.edu.cn (S.J.); yongchaodyc@126.com (Y.D.); 15895571861@163.com (R.Z.); jiangyiguo0515@126.com (Y.J.); 2 State Key Laboratory of Pharmaceutical Biotechnology, School of Life Sciences, Nanjing University, Nanjing 210023, China; 522024300043@smail.nju.edu.cn

**Keywords:** sleep deprivation, edaravone, cognitive impairment, oxidative stress, mice

## Abstract

**Background:** Sleep deprivation (SD) is a common condition affecting many people in modern life. Edaravone (Eda) is a neuroprotective drug, but whether it can improve cognitive impairment caused by SD remains unclear. **Methods:** Animals received oral gavage doses of Eda (10, 20, and 40 mg/kg) for 8 and 5 days before and during SD. Starting from the 6th day, a modified multiple platform model was used to produce SD in mice for 72 h. The Lashley III maze was used for evaluating spatial learning and memory. Malondialdehyde (MDA) in the hippocampus and serum corticosterone were assessed. Total antioxidant capacity (TAC) and the activity of the enzymes glutathione peroxidase (GPx) and superoxide dismutase (SOD) were measured. Growth-associated protein 43 (GAP-43), synapsin 1 (SYN-1), post-synaptic density-95 (PSD-95), synaptophysin (SYP), and signs of inflammation were detected using Western blotting. **Results:** SD caused cognitive impairment, whereas Eda pretreatment warded off such an effect. While serum corticosterone levels rose with SD as well, they decreased in SD mice that received Eda (*p* < 0.05). Moreover, Eda normalized the SD-induced decline in hippocampal activity of SOD and GPx, lowered MDA levels, and elevated TAC (*p* < 0.01). Additionally, the hippocampal levels of GAP-34, SYP, SYN-I, and PSD-95 were elevated, while IL-1β and tumor necrosis factor α (TNF-α) were lowered following Eda pretreatment (*p* < 0.05). **Conclusions:** SD caused memory impairment; however, pretreatment with Eda improved memory by upregulating synaptic proteins in the hippocampus and having anti-inflammatory and antioxidant effects.

## 1. Introduction

Sleep insufficiency represents a global health concern with profound implications for neurological functioning. Previous studies have established sleep deprivation (SD) as a significant contributing factor to the pathogenesis and exacerbation of various psychiatric and neurological disorders [1]. Robust evidence also demonstrates sleep’s essential role in learning and memory consolidation processes, with deficiencies in either sleep duration or quality consistently manifesting as impairments in these critical cognitive functions [2].

Sleep facilitates optimal cognitive performance by preparing neural circuits for information acquisition, consolidation within existing memory networks, and integrative processing. Conversely, SD compromises numerous cognitive domains, including perceptual processing, memory formation, and executive functioning [3]. Recent investigations have revealed that SD specifically undermines hippocampal integrity—a brain region instrumental for memory encoding and neurocognitive consolidation. Protracted SD induces deleterious neurochemical alterations within hippocampal circuitry, characterized by elevated concentrations of lipid peroxidation products and heightened oxidative stress parameters, presumably resulting from disruption of redox homeostasis mechanisms [4]. Moreover, sleep-deprived animal models exhibit significantly increased levels of pro-inflammatory cytokines—particularly tumor necrosis factor-α (TNF-α) and interleukin-1β (IL-1β)—within specific hippocampal subregions, indicating differential vulnerability to inflammatory processes following sleep restriction [5].

Compelling evidence indicates substantial neuronal damage consequent to SD, underscoring the clinical imperative for neuroprotective pharmacological interventions in chronically sleep-deprived individuals. Over recent decades, researchers have investigated diverse compounds, including vitamins C [6] and E [7], melatonin [8], and caffeine [9], for their potential to mitigate SD-induced oxidative stress, neuroinflammation, and resultant memory deficits. Among these therapeutic candidates, edaravone (Eda) has emerged as a promising neuroprotective agent with demonstrated anti-oxidative and anti-inflammatory properties [10].

The neuroprotective efficacy of Eda extends across multiple neurological conditions, including peripheral diabetic neuropathy [11] and experimental models of Alzheimer’s disease [12]. Mechanistically, Eda modulates serum corticosterone homeostasis and attenuates neuroinflammatory signaling cascades within prefrontal-hippocampal networks, as evidenced by downregulated protein expression of TNF-α, NF-κB, and IL-1β. Additionally, Eda suppresses reactive oxygen species (ROS) and malondialdehyde (MDA) accumulation while concurrently enhancing endogenous antioxidant enzyme activities [13].

Given these promising attributes, our investigation sought to systematically evaluate the potential neuroprotective effects of Eda against SD-induced neuroinflammation, oxidative stress, and consequent cognitive impairments in murine experimental models.

## 2. Material and Methods

### 2.1. Animals

We purchased fifty male C57BL/6 young-adult mice (8 weeks old) weighing 18 ± 2 g from Beijing Vital River Laboratory Animal Technology Co., Ltd., Beijing, China. Animals were housed under standard laboratory conditions at 25 ± 1 °C and 60% humidity. They were also given food and tap water and kept on a 12 h light/dark cycle. The light intensity was specified as the standard laboratory light intensity, and the light period was from 8 a.m. to 8 p.m. every day, and the dark period was from 8 p.m. to 8 a.m. the next day. Before beginning the experiment, the mice were acclimated to laboratory settings.

### 2.2. Sleep Deprivation Protocol and Drug Administration

**Figure 1:**
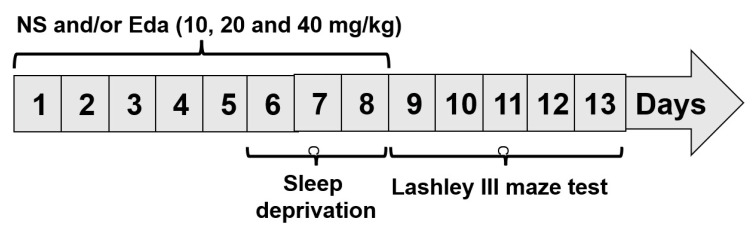
Experimental design.

The animals were divided into two groups at random: the SD (n = 40) and wide platform (WP) (n = 10). The protocol entails positioning an acrylic chamber measuring 42 cm × 30 cm × 20 cm, equipped with 12 circular platforms, which housed five mice. Large-diameter platforms (6 cm) were implemented in the WP cohort to facilitate sustained murine sleep while preventing aqueous immersion. On the other hand, a revised multiple-platform approach was employed to subject the mice in the SD Group to SD for a period of 72 h. Animals were positioned 1 cm deep and 1.5 cm in diameter beneath the platform’s upper surface. The experimental subjects retained locomotor capacity within the hydrodynamic environment through saltatory transitions between elevated platforms. In addition, the animals were provided with aqueous sustenance and solid food pellets via the cage cover [14]. Four subgroups were created out of the SD group: Eda 10, Eda 20, and Eda 40, in addition to normal saline receiving (NS). Based on previous studies and references [15], the animals underwent a pre-treatment regimen involving administration of NS and 10, 20, and 40 mg/kg Eda, with the treatment spanning a total duration of 8 days. It commenced 5 days before the onset of the 72-h SD period, and the drugs were delivered via oral gavage (Figure 1).

**Figure 2:**
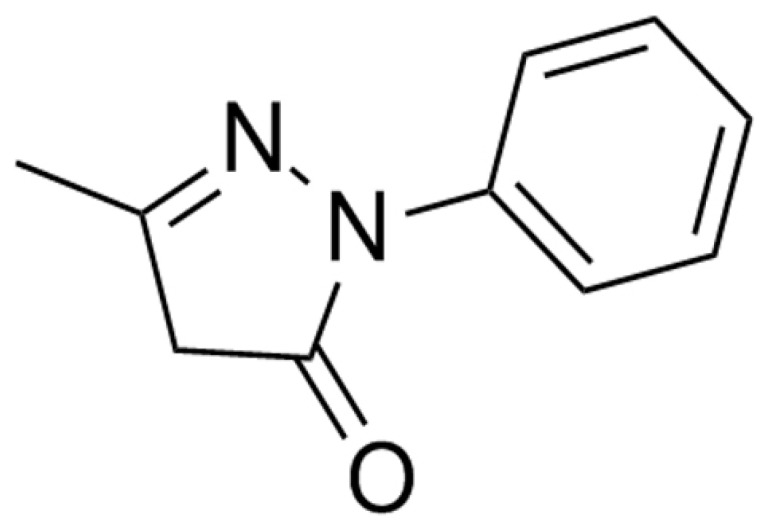
Chemical structures of edaravone.

Edaravone (Xian Lyphar Biotech Co., Xi’an, China) was reconstituted in sterile 0.9% sodium chloride solution under aseptic conditions. The chemical structure of edaravone is shown in Figure 2.

### 2.3. Lashley III Maze

The Lashley III maze paradigm was employed to assess spatial memory consolidation and associative learning through distal visual cues and appetitive reinforcement. The maze’s lateral boundaries and overhead structure were fabricated from either opaque or translucent poly (methyl methacrylate) panels. This maze consisted of four connected runways measuring 45 cm in length, 7 cm in height, and 5 cm in width; a start box measuring 8 cm by 9.5 cm by 7 cm; and a goal box measuring 19.5 cm in length, 7 cm in height, and 5 cm in width that held a food reward. Thirty minutes prior to the acclimatization test, mice that had not consumed any food were transferred to the testing room. Each animal was placed separately in the start box for a duration of 15 s at the onset of the test. A 4 × 4 cm Plexiglas sliding door, 11 cm from the maze’s outside borders, was opened, allowing the object to move through the maze and eventually arrive at the goal box. Over a 5-day acquisition period, mice underwent daily training sessions in the light phase, with all behavioral parameters digitally captured via overhead video tracking system. Three neurobehavioral endpoints were analyzed: (1) error frequency (incorrect arm entries), (2) goal-seeking latency (time from maze entry to target quadrant), and (3) cognitive performance index (ratio of target quadrant occupancy duration to total trial time across five consecutive training days) [16].

### 2.4. Biochemical Testing [17]

Using a ketamine and xylazine combination (90 and 10 mg/kg, respectively), animals were anesthetized following behavioral testing. Serum was collected via transcardial phlebotomy and immediately processed at low temperatures. Whole blood specimens underwent centrifugation at 1000× *g* for 10 min under 4 °C conditions to separate the supernatant fraction. It did not take long to separate the brains from the skull. After being taken out, the hippocampi were submerged in liquid nitrogen. Prior to analysis, all samples were gathered and kept at 80 °C.

### 2.5. Serum Concentration of Corticosterone

Blood was collected from the retroorbital vein of each animal in serum vacutainers. The samples were allowed to clot at room temperature for 30 min and then centrifuged at 2500× *g* for 15 min. Serum levels of corticosterone were measured using an ELISA kit (catalogue number ADI-901-097, EnzoLife Sciences, Bruxelles, Belgium). Samples were diluted 1:40 before running. The reaction was carried out in duplicate according to the kit instructions, and the average absorbance of the plate was determined using a plate reader [18].

### 2.6. Lipid Peroxidation and Antioxidant Activity Assessment [19]

Samples of frozen hippocampi were homogenized in a 1.5% KCl solution and centrifuged for 10 min at 4 °C at 1000 rpm. Thiobarbituric acid-reactive compounds were used to quantify the estimated MDA levels in the hippocampus supernatant (TBARS). At a wavelength of 535 nm, the absorbance of the reaction mixture was measured. The brain’s MDA concentrations were expressed as nmol/mg of protein.

The total antioxidant capacity (TAC) was assessed using the Randox total antioxidant status kit (NX2332, Randox Laboratories Ltd., County Antrim, UK), in which antioxidants were used in accordance with antioxidant capacity and concentration to decolorize the 2,2′-azinobis [3-ethylbenzothiazoline-6-sulfonic acid] radical cation (ABTS^+^). The absorbance at 600 nm was used to measure this color change.

The superoxide dismutase (SOD) activity was assessed employing the RASOD kit (SD125, Randox) in line with the protocol outlined by Delmas-Beauvieux et al. [20] To extract the necessary solutions and the supernatant, the hippocampus tissue was homogenized. Using spectrophotometry, absorbance was measured at 505 nm (Pharmacia Biotech, Sofia, Bulgaria, UK).

Glutathione peroxidase (GPx) activity was determined in accordance with the methodological framework established by Paglia and Valentine, employing the RANSEL assay system (RS505, Randox). Homogenization of cryopreserved hippocampal tissues was conducted with 50 mM phosphate buffer supplemented with 0.5 mM EDTA (pH 7.5). The homogenates underwent a 15 min, 10,000× *g* centrifugation at 4 °C. Using a spectrophotometer, the absorbance of the supernatant at 340 nm decreased at 37 °C. SOD and GPx activity data are shown as U/mg of sample protein.

### 2.7. Western Blotting

Western blot analysis was used to assess the levels of post-synaptic density-95 (PSD-95), growth-associated protein 43 (GAP-43), synapsin I (SYN-I), and synaptophysin (SYP) in the hippocampus. For every group, three samples of hippocampus tissue were used. After blending brain tissue in 100 μL of chilled RIPA lysis buffer with a protease inhibitor cocktail, the mixture was centrifuged at 12,000× *g* for 15 min at 4 °C. The Bradford method was used to determine the amount of protein in the supernatant. Following a 10 min boil, the samples were mixed with Sample Loading Buffer 2X (Sigma, St. Louis, MO, USA). Proteins were deposited onto polyvinylidene difluoride membranes (Roche, Hertfordshire, UK) after being separated by 12.5% polyacrylamide gel electrophoresis. Major antibodies of SYN-1 (1:500, ab254349), GAP43 (1:500, ab75810), PSD-95 (1:500, ab18258), SYP (1:500, ab32127), and β-actin (1:500, ab8226) were incubated at 4 °C for 24 h on the membranes. Every antibody was acquired from Abcam, located in Cambridge, Massachusetts, USA. After three rounds of washing with phosphate-buffered saline, the membrane was incubated for two hours at room temperature with an anti-rabbit secondary antibody (1:5000, ab150077) coupled with horseradish peroxidase. The membranes were exposed to autoradiography film (Kodak, Rochester, NY, USA) to view the signals after being placed in a strengthened chemiluminescence detection solution (Amersham, Little Chalfont, UK). The intensity of each band’s signal was determined using ImageJ 1.62 software (National Institutes of Health, Bethesda, MD, USA), and the outcomes were normalized to the corresponding internal control.

### 2.8. Statistical Analysis

Data were analyzed using GraphPad Prism 9, and the results are expressed as the mean standard error of the mean (S.E.M.). To make sure the data were normal, the Shapiro–Wilk test was run. To find statistically significant differences between the groups, post hoc Tukey’s tests and one- or two-way analysis of variance (ANOVA) were used. In the two-way analysis (Lashley III Maze), the two variables of interest were the day and the group; on the other hand, the outcomes were compared between groups on the corresponding days. Tukey correction was used for all multiple comparisons, Western blot data were validated by three independent experiments, and batch effects were included as a random variable for mixed-effects model analysis. Statistical significance was determined at *p* < 0.05 for all comparisons.

## 3. Results

### 3.1. Effect of Edaravone Treatment on the Learning and Memory of SD Mice

**Figure 3:**
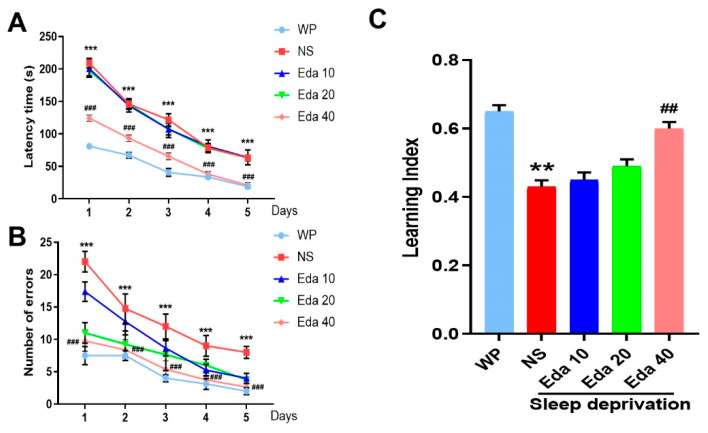
Eda alleviated the symptoms of sleep deprivation-induced memory disorder. Mice were accepted sleep deprivation for 3 days and behavioral test for 5 days. Mice were intragastric administration with Eda (10, 20, 40 mg/kg) once a day. Latency time (**A**), number of errors (**B**), and learning index (**C**) were measured. All data are expressed as the mean ± SD (n = 10). ** *p* < 0.01, *** *p* < 0.001 vs. WP, ^##^ *p* < 0.01, ^###^ *p* < 0.001 vs. Eda group.

A two-way ANOVA demonstrated the substantial effects of group, but did not indicate group–day interaction, when the delay time was assessed throughout the course of five training days for each group. The average time taken to reach the goal box was significantly distinct between the WP group and the NS + SD group on the fourth (*p* < 0.01) and fifth (*p* < 0.001) days, as depicted in Figure 3A. Additionally, significant performance differences emerged between treatment groups during the training phase. Animals in the SD + Eda 20 group displayed significantly improved performance relative to the SD + NS group on days 1 (*p* < 0.001) and 2 (*p* < 0.05). Most notably, the SD + Eda 40 group consistently outperformed the SD + NS group across all five training days (*p* < 0.001, *p* < 0.001, *p* < 0.01, *p* < 0.001, and *p* < 0.05, respectively), suggesting a dose-dependent amelioration of sleep deprivation-induced performance deficits. Furthermore, a two-way ANOVA showed that group and day had a significant impact on the total number of errors throughout the course of the five training days, but not group–day interaction. On days 1 through 5 (all days, *p* < 0.001), the mean number of errors in the NS + SD group was noticeably higher than in the WP group. Relative to the comparison days in the SD + NS group, the number of errors on days 1–5 of training was reduced in the SD + Eda 20 (*p* < 0.001, *p* < 0.01, *p* < 0.001, *p* < 0.001, and *p* < 0.05, respectively, across days 1–5) and SD + Eda 40 groups (*p* < 0.001, for all days, Figure 3B).

A significant difference (n = 10) in alternation between the groups was found by the one-way ANOVA findings. NS + SD mice had a worse learning index than WP mice (*p* < 0.05), as Figure 3C illustrates. On the other hand, during the 5-day trials, Eda at a dose of 40 mg/kg (*p* < 0.05) raised the mean values of the learning index.

### 3.2. Serum Corticosterone Levels

**Figure 4:**
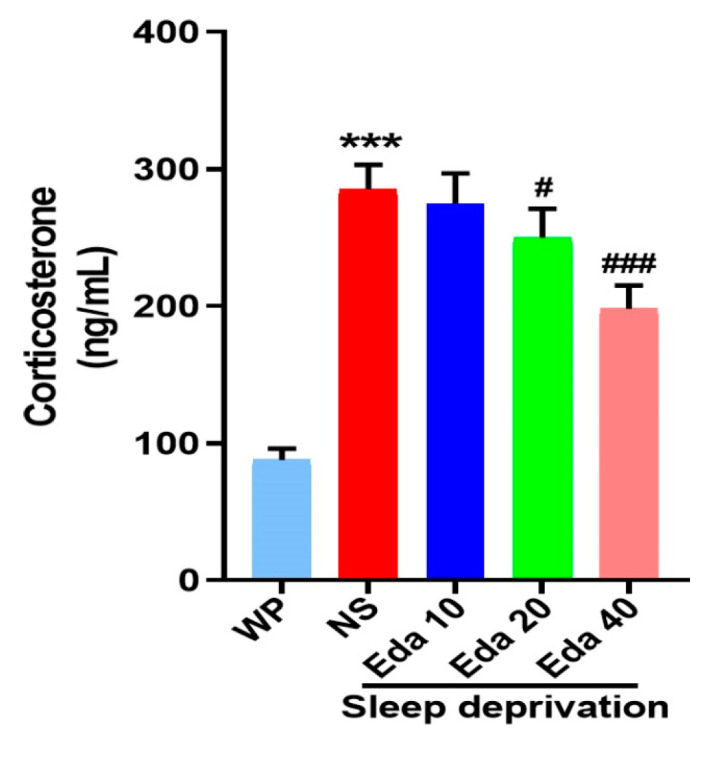
The protect effect of Eda on serum corticosterone levels. All data are expressed as the mean ± SD (n = 6). *** *p* < 0.001 vs. WP, ^#^ *p* < 0.05, ^###^ *p* < 0.001 vs. Eda group.

The experimental serum corticosterone concentrations were quantified at the study endpoint. Intergroup differences in serum corticosterone concentrations demonstrated statistical significance, as evidenced by one-way ANOVA (n = 6). Figure 4 indicates that while acute SD caused a significant increase in serum corticosterone levels when compared with those in the WP group (*p* < 0.001), Eda administration remarkably decreased corticosterone levels at doses of 20 mg/kg (*p* < 0.05) and 40 mg/kg (*p* < 0.001).

### 3.3. MDA Levels

**Figure 5:**
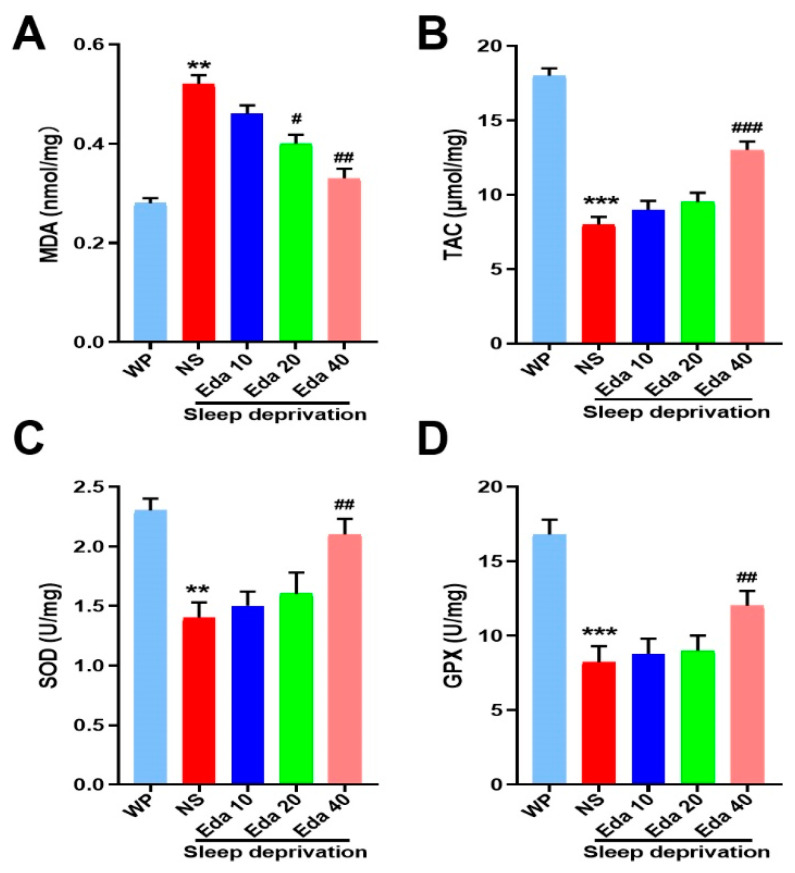
The protect effect of Eda on (**A**) MDA levels, (**B**) TAC levels, (**C**) SOD activity, and (**D**) GPx activity. All data are expressed as the mean ± SD (n = 8). ** *p* < 0.01, *** *p* < 0.001 vs. WP, ^#^ *p* < 0.05, ^##^ *p* < 0.01, ^###^ *p* < 0.001 vs. Eda group.

One-way ANOVA showed that there was a significant difference (n = 8) in MDA levels between the groups. According to our findings, animals’ MDA levels increased significantly after SD when compared to WP animals (*p* < 0.001). However, as compared to SD mice that were not treated, Eda treatment at a dose of 40 mg/kg reduced MDA levels (*p* < 0.001, Figure 5A).

### 3.4. Antioxidant Status

A marked discrepancy in TAC concentrations (n = 8) was observed among the groups. The findings revealed that exposure to SD resulted in a substantial decrease in TAC levels (*p* < 0.05). However, Eda 40 mg/kg therapy corrected the loss in hippocampus TAC caused by SD (*p* < 0.05, Figure 5B). Additionally, a noteworthy distinction was discovered in the groups’ SOD (n = 8) and GPx (n = 8) activities. When compared to the control group, the activity of SOD and GPx was significantly reduced by SD (*p* < 0.001). On the seventh day, SOD and GPx activity rose at a dose of 40 mg/kg Eda in comparison to the NS + SD group (*p* < 0.05, Figure 5C,D).

### 3.5. IL-1β and TNF-α

**Figure 6:**
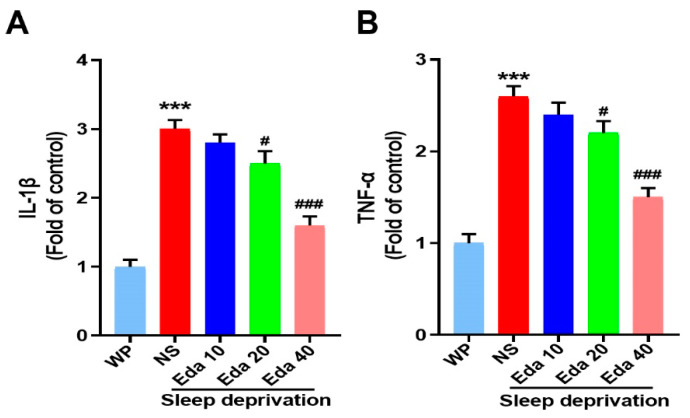
The protect effect of Eda on (**A**) IL-1β and (**B**) TNF-α levels were measured by ELISA. All data are expressed as the mean ± SD (n = 6). *** *p* < 0.001 vs. WP, ^#^ *p* < 0.05, ^###^ *p* < 0.001 vs. Eda group.

Significant discrepancies in the protein levels of TNF-α (n = 6) and IL-1β (n = 6) were observed among the groups. In the hippocampal regions of mice exposed to acute SD, as illustrated in Figure 6A, IL-1β expression was higher than in WP mice (*p* < 0.001). In contrast to the NS + SD group, pretreatment with Eda dramatically reduced IL-1β expression at dosages of 20 and 40 mg/kg (*p* < 0.05 and *p* < 0.001, respectively). Additionally, compared to the WP group, exposure to SD significantly raised TNF-α levels (*p* < 0.001, Figure 6B). In contrast, TNF-α levels were significantly reduced by Eda pretreatment at all dosages (*p* < 0.05, 20 mg/kg, and *p* < 0.001 for 40 mg/kg) when compared to the NS + SD group.

### 3.6. Synaptic Integration Markers

**Figure 7:**
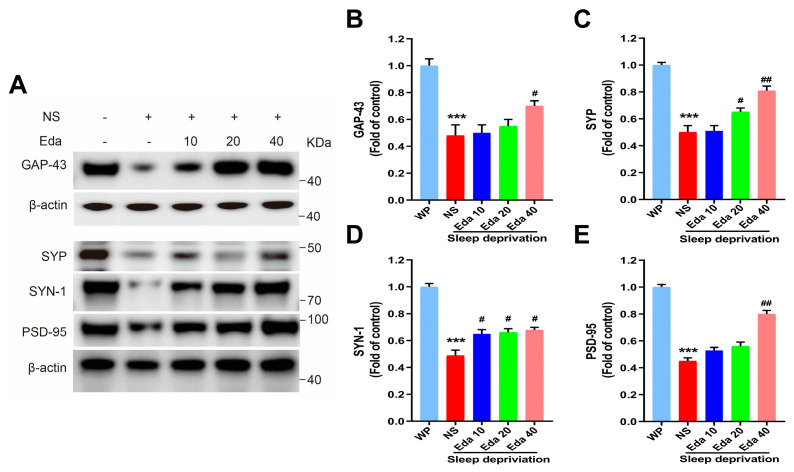
The Eda inhibits Synaptic integration-related proteins in hippocampal tissues. Representative Western blot images show the relative expressions of related proteins in the groups (**A**). The protein levels (normalized) of GAP-43 (**B**), SYP (**C**), SYN-1(**D**) and PSD-95 (**E**) in hippocampal tissues.

One-way ANOVA statistical analysis demonstrated statistically significant differential expression of GAP-43, SYP, SYN-1, and PSD-95 across experimental cohorts (n = 6). As illustrated in Figure 7A, a significant reduction in hippocampal GAP-43 protein expression was noted after SD in comparison with the WP group (*p* < 0.001). A 40 mg/kg dose of Eda therapy may partially (*p* < 0.05) restore GAP-43 levels. When comparing the hippocampal SYP levels of SD mice to those of the WP group, Figure 7B demonstrates that acute SD significantly decreased these levels (*p* < 0.001). On the other hand, pretreatment of sleep-deprived (SD) animals with Eda at dosages of 20 and 40 mg/kg significantly elevated synaptophysin (SYP) levels (*p* < 0.05 and *p* < 0.001, respectively) compared to untreated SD animals (Figure 7C). Notably, SD led to a substantial downregulation of SYN-1 and PSD-95 expression relative to the well-rested (WP) cohort (*p* < 0.001). Administration of Eda reversed these detrimental effects, showing a dose-dependent upregulation of SYN-1 across all tested doses (*p* < 0.05) and a selective enhancement of PSD-95 specifically at 40 mg/kg (*p* < 0.01), when compared to saline (NS)-treated SD counterparts (Figure 7D,E).

## 4. Discussion

Sleep plays a critical role in maintaining mental, emotional, and physiological health and facilitates waste clearance from the brain [21]. It also serves essential functions in brain plasticity and memory consolidation. Sleep deprivation (SD), particularly prevalent among elderly populations, represents a growing burden on global healthcare resources and is associated with cognitive impairment [22]. SD impairs hippocampal function and memory by elevating neuroinflammation, oxidative stress, and lipid peroxidation while concurrently undermining synaptic plasticity [23]. Additionally, SD is associated with reduced cerebral blood flow and metabolic activity in key brain regions, including the thalamus, parietal cortex, and prefrontal cortex [24].

The results of the present study demonstrated that SD was associated with elevated levels of corticosterone in the blood, suggesting that SD stimulates the hypothalamic–pituitary–adrenal (HPA) axis. Consistent with these findings, two prior studies reported increased corticosterone levels in the serum of animals subjected to SD [25]; moreover, emerging evidence suggests that SD-induced hyperactivity of the amygdala-prefrontal cortex circuit may serve as a shared neural substrate for both anxiety and cognitive decline [26]. Adrenocorticotropic hormone (ACTH), cortisol production, and HPA axis activity are all associated with insomnia. In contrast, the administration of glucocorticoids has been found to result in heightened alertness and sleeplessness [27], which is driven by significant upregulation of genes regulating the HPA axis, particularly proopiomelanocortin and corticotropin-releasing factor receptor 1. Consequently, elevated cortisol levels adversely impact cognitive function and contribute to the pathophysiology of Alzheimer’s disease. Notably, mice subjected to SD did not exhibit elevated corticosterone levels in their serum when pretreated with Eda (20 or 40 mg/kg). A prior study revealed that pretreatment with 10 mg/kg Eda reduced corticosterone levels in the serum of Albino Swiss mice exhibiting depressive- and anxiety-like behaviors induced by restraint stress [28].

Both acute and chronic SD have been shown to increase oxidative stress, as evidenced by elevated malondialdehyde (MDA) levels and reduced activities of antioxidant enzymes such as superoxide dismutase (SOD) and glutathione peroxidase (GPx) in the hippocampus and other brain regions [29]. Oxidative stress, which damages synapses and neurons, contributes to cognitive impairment. In the present study, a 72 h acute SD regimen was found to modify total TAC levels and disrupt antioxidant enzyme defense mechanisms in the hippocampus. Furthermore, treatment with Eda was associated with enhanced SOD and GPx activity in the hippocampus, increased TAC levels, and reduced MDA levels. These findings underscore Eda’s antioxidant properties and demonstrate its potential as a neuroprotective agent at a dose of 40 mg/kg by strengthening the body’s defense against free radical damage. The antioxidative effects of Eda have been previously documented in the brain and other rodent studies [30].

TNF-α and IL-1β are two major inflammatory cytokines that are upregulated in response to increased oxidative stress, as they activate the NF-κB signaling pathway. Numerous studies have shown that in both human and animal models, SD increases markers of oxidative stress and triggers inflammatory responses [31]. In addition, previous studies have found increased concentrations of IL-1β and TNF-α in systemic circulation as well as in specific brain regions following SD [32]. Our findings showed increased expression of TNF-α and IL-1β in the brains of mice after SD, as well as overactivation of the HPA axis and oxidative stress. Moreover, there is a strong correlation between increased brain inflammation and neuronal loss, as well as cognitive decline. We demonstrated that SD-induced IL-1β and TNF-α expression was significantly reduced by Eda administration. Furthermore, previous studies have shown that Eda administration at doses of 150 and 200 mg/kg suppresses the expression of NF-κB, TNF-α, and IL-1β in the hippocampus and prefrontal cortex of mice exposed to chronic restraint stress [33]. Eda has anti-inflammatory properties, including the generation of nitric oxide, suppression of cyclooxygenase-2, and inhibition of IL-1β, TNF-α, and IFN-γ. These properties have been demonstrated both in vitro and in vivo, and Eda also increases the synthesis of IL-2 and IL-10.

There is mounting research that shows SD disrupts hippocampus-dependent memory. Preclinical and clinical studies suggest that SD-induced memory impairment may stem from oxidative stress and neuroinflammation. Acute SD lasting 24–72 h has been shown to impair short- and long-term memory mediated by hippocampal circuitry. In this study, animals subjected to 72 h of SD exhibited reduced spatial reference memory during the Lashley task compared to the WP group. Consistent with our findings, numerous studies have demonstrated cognitive impairments in SD using various learning and memory assessments, such as the eight-box task, radial arm water maze, contextual fear conditioning, and novel object recognition. Conversely, additional investigations have documented that SD exerts no detectable effect on mnemonic processes, potentially ascribed to methodological divergences in task complexity, sleep phase, memory modalities assessed, and SD protocols [34]. The long-term effects of SD and other forms of psychological stress have been examined in several research studies. In male rats, Bersagliere et al. observed aberrant EEG waves on four recovery days following a 24 h period of SD [35]. Furthermore, Fifel et al. revealed pathological modifications in the functional dynamics of neurons within the paraventricular nucleus, arcuate nucleus, lateral hypothalamus, and mammillary bodies [36]. Wu et al. proposed that the dysregulation of circadian rhythms and homeostatic processes represents a key persistent consequence of SD [37].

Learning and memory are profoundly modulated by GAP-43, which regulates presynaptic neuronal maturation, neurotransmission, and neuritogenesis. PSD-95 is a key modulator of synaptic strength and plasticity. Additionally, SYP functions in neurotransmitter release, synaptic vesicle biogenesis, and synaptic plasticity. According to Yu et al., SD causes synaptic degradation in the hippocampal regions by reducing PSD-95, SYP, and SYN-1 levels. Another study found that after 8 and 48 h of SD, SYN-1 mRNA expression in the hippocampus was downregulated [38]. Basheer et al. demonstrated altered synaptic efficacy and cytoskeletal proteins following SD, showing decreased expression of proteins involved in presynaptic vesicle and postsynaptic receptor trafficking, as well as long-term potentiation (SNAP25, N-ethylmaleimide-sensitive factor [NSF] attachment protein, vesicle-associated membrane protein [VAMP]), along with functional inactivation of amphiphysin I [39]. Accordingly, after 72 h of SD, our findings showed a decreased density of synaptic proteins (GAP-3, PSD95, SYP, and SYN-1) compared to the WP group. However, therapy with Eda attenuated this decrease in synaptic protein levels. The improvement in spatial reference memory deficits observed in this experiment may be attributed to the increase in synaptic protein levels following Eda treatment after SD.

## 5. Conclusions

The enhancement of spatial reference memory deficit observed in this experiment may be ascribed to the elevation of synaptic protein levels in conjunction with SD following Eda treatment. Furthermore, in the hippocampal regions of SD mice, pretreatment with Eda decreased neuroinflammation, oxidative stress, and memory impairment while restoring presynaptic and postsynaptic protein levels. In the future, Eda is expected to be used in the dimensional treatment of neuropsychiatric syndromes.

## Data Availability

All data used to support this study are available from the corresponding author upon request.
